# A New Method for Visualizing Drusen and Their Progression in Flood-Illumination Adaptive Optics Ophthalmoscopy

**DOI:** 10.1167/tvst.10.14.19

**Published:** 2021-12-20

**Authors:** Ethan A. Rossi, Nathaniel Norberg, Chiara Eandi, Celine Chaumette, Saloni Kapoor, Laura Le, Valerie C. Snyder, Joseph N. Martel, Josselin Gautier, Kiyoko Gocho, Kunal K. Dansingani, Jay Chhablani, Angelo Arleo, Sarah Mrejen, José-Alain Sahel, Kate Grieve, Michel Paques

**Affiliations:** 1 Department of Ophthalmology, University of Pittsburgh School of Medicine, Pittsburgh, PA, USA; 2 Department of Bioengineering, University of Pittsburgh Swanson School of Engineering, Pittsburgh, PA, USA; 3 McGowan Institute for Regenerative Medicine, University of Pittsburgh, Pittsburgh, PA, USA; 4 Sorbonne Université, INSERM, CNRS, Institut de la Vision, 17 rue Moreau, F-75012 Paris, France and CHNO des Quinze-Vingts, INSERM-DGOS CIC 1423, 28 rue de Charenton, F-75012 Paris, France; 5 Department of Surgical Science, University of Torino, Turin, Italy

**Keywords:** drusen, age-related macular degeneration, adaptive optics, image analysis

## Abstract

**Purpose:**

Drusen are dynamic sub-RPE deposits that are risk factors for late-stage age-related macular degeneration (AMD). Here we show a new imaging method using flood-illumination adaptive optics (FIAO) that reveal drusen with high contrast and resolution.

**Methods:**

A fovea-centered 4° × 4° FIAO image and eight surrounding images with gaze displaced by ±2° vertically and horizontally were acquired. Clinical color fundus and spectral-domain optical coherence tomography were acquired for clinical grading and comparison. Custom software registered overlapping FIAO images and fused the data statistically to generate a fovea-centered 4° × 4° gaze-dependent image. Our dataset included 15 controls (aged 31–72) and 182 eyes from 104 AMD patients (aged 56–92), graded as either normal aging (n = 7), and early (n = 12), intermediate (n = 108) and late AMD (n = 42); 27 had subretinal drusenoid deposits (SDDs), and 83 were imaged longitudinally.

**Results:**

No gaze varying structures were detected in young eyes. In aging eyes with no evidence of age-related changes, putative drusen <20 µm in diameter were visible. Gaze-dependent images revealed more drusen and many smaller drusen than visible in color fundus images. Longitudinal images showed expansion and fusion of drusen. SDDs were lower contrast, and RPE atrophy did not yield a consistent signal.

**Conclusions:**

Gaze-dependent imaging in a commercially available FIAO fundus camera combined with image registration and postprocessing permits visualization of drusen and their progression with high contrast and resolution.

**Translational Relevance:**

This new technique offers promise as a robust and sensitive method to detect, map, quantify, and monitor the dynamics of drusen in aging and AMD.

## Introduction

Drusen, a phenotypic hallmark of early age-related macular degeneration (AMD), are deposits of extracellular material between the basal lamina of the retinal pigment epithelium (RPE) and the inner collagenous layer of Bruch's membrane.^1^ Although their pathophysiology remains to be fully elucidated, they are considered risk factors for both type 1 macular neovascularization and geographic atrophy.^2^ Drusen often grow with time, and it is commonly assumed that drusen size is correlated to the risk of evolution to late stages of AMD. Small drusen (<63 µm), also referred to as “hard” drusen, may be nonpathological, because many young subjects may have some small drusen.^3^ The presence of intermediate (63–125 µm) and large (>125 µm) drusen is more ominous, especially when associated with pigmentary changes.^4^ Large drusen have fuzzy margins in color fundus photography and are often referred to as “soft” drusen. Another type of age-related deposition are subretinal drusenoid deposits (SDDs) that reside above the RPE^5^ and whose presence are also a risk factor for late AMD.^6^^,^^7^

Because the amount and size of drusen are positively correlated with progression to visually threatening late-stage AMD, there is strong interest in high precision imaging of drusen. Color fundus photography was used in the AREDS studies to grade AMD stage and quantify drusen.^8^ Optical coherence tomography (OCT) can determine drusen extent in cross-section or volumetric reconstructions,^9^^,^^10^ along with associated microstructural changes, although visualization of the smallest drusen may challenge the capabilities of clinical-grade imaging systems.

The use of high-resolution imaging such as adaptive optics ophthalmoscopy (AOO) has provided new insight into AMD at the cellular scale.^11^^–^^22^ However, drusen have presented challenges for AOO imaging, because they generally show up as poorly delineated hyper-reflective spots.^3^^,^^15^ Drusen can displace photoreceptor outer segments and interfere with their pointing direction, decreasing their visibility in adaptive optics scanning light ophthalmoscopy (AOSLO). This can lead to difficulties in the interpretation of confocal AOSLO photoreceptor images in the context of drusen.^3^^,^^18^ Confocal AOSLO has also been used to evaluate the impact of different subtypes of SDDs on photoreceptor imaging.^20^

Flood-illumination adaptive optics (FIAO) imaging can also resolve drusen, yet they often have relatively low contrast across much of their extent.^15^ We incidentally observed that the contrast of drusen can be asymmetric in FIAO, with some margins of a druse appearing higher contrast than others. We subsequently noted that such contrast variability was strongly related to gaze position. As gaze position changes, it alters the relative position of the drusen within the field of view of the imaging system and thus the location of the drusen with respect to the illumination in FIAO. Here we capitalized on this variability to develop an image processing routine to enable visualization of the margins and extent of drusen with high contrast. We show that drusen contrast can be enhanced by registering FIAO images from different gaze positions and fusing them into a composite image with simple statistical operations.

## Methods

### Participants

Patients with AMD (n = 104 [69 female]; aged 56–92; mean: 74; SD: 7.8) and controls (n = 15; aged 31–72) were recruited from the Quinze-Vingts National Ophthalmology Hospital and the University of Pittsburgh Department of Ophthalmology. Written informed consent was obtained from all participants after an explanation of experimental procedures and risks both verbally and in writing. All experiments were approved by the institutional review boards of the University of Pittsburgh and the Quinze-Vingts National Ophthalmology Hospital and adhered to the tenets of the Declaration of Helsinki. All light levels used were well below the limits specified by the American National Standard Institute and International Standardization Organization.

Participants had refractive errors between −6 and +2 diopters, best corrected visual acuity of at least 20/60 on the imaged eye and sufficient clarity of the ocular media and lens for clinical imaging and FIAO. Exclusion criteria included conditions that affected a participant's ability to sit in a chin rest for imaging, allergy, or susceptibility to acute glaucoma from mydriatics, choroidal neovascularization, nystagmus, diabetic retinopathy, or other macular diseases such as central serous chorioretinopathy. Eighty-three eyes were imaged longitudinally at multiple timepoints at variable intervals ranging from weekly to annually to evaluate reproducibility and evolution of drusen over time.

### Image Acquisition

Clinical imaging included color fundus photography (Topcon TRC-501X; Topcon Optical Company, Tokyo, Japan), scanning laser ophthalmoscopy (SLO) and spectral domain optical coherence tomography (SD-OCT; Spectralis HRA+OCT; Heidelberg Engineering, Heidelberg, Germany). Several SD-OCT volume scans were obtained, including (1) coarse macula 20° × 20° (25 sections, automatic real time averaging (ART): 9 frames, volume scan); (2) dense macula 15° × 5° (131 sections, ART: 0[en face]); and (3) vertical and horizontal lines (macula centered, ART: 24). For each SD-OCT scan, a “quality” value of at least 8 was required. The Spectralis SLO was used to acquire infrared reflectance SLO images.

### FIAO Gaze-Dependent Image Acquisition Procedure

**Figure 1.**
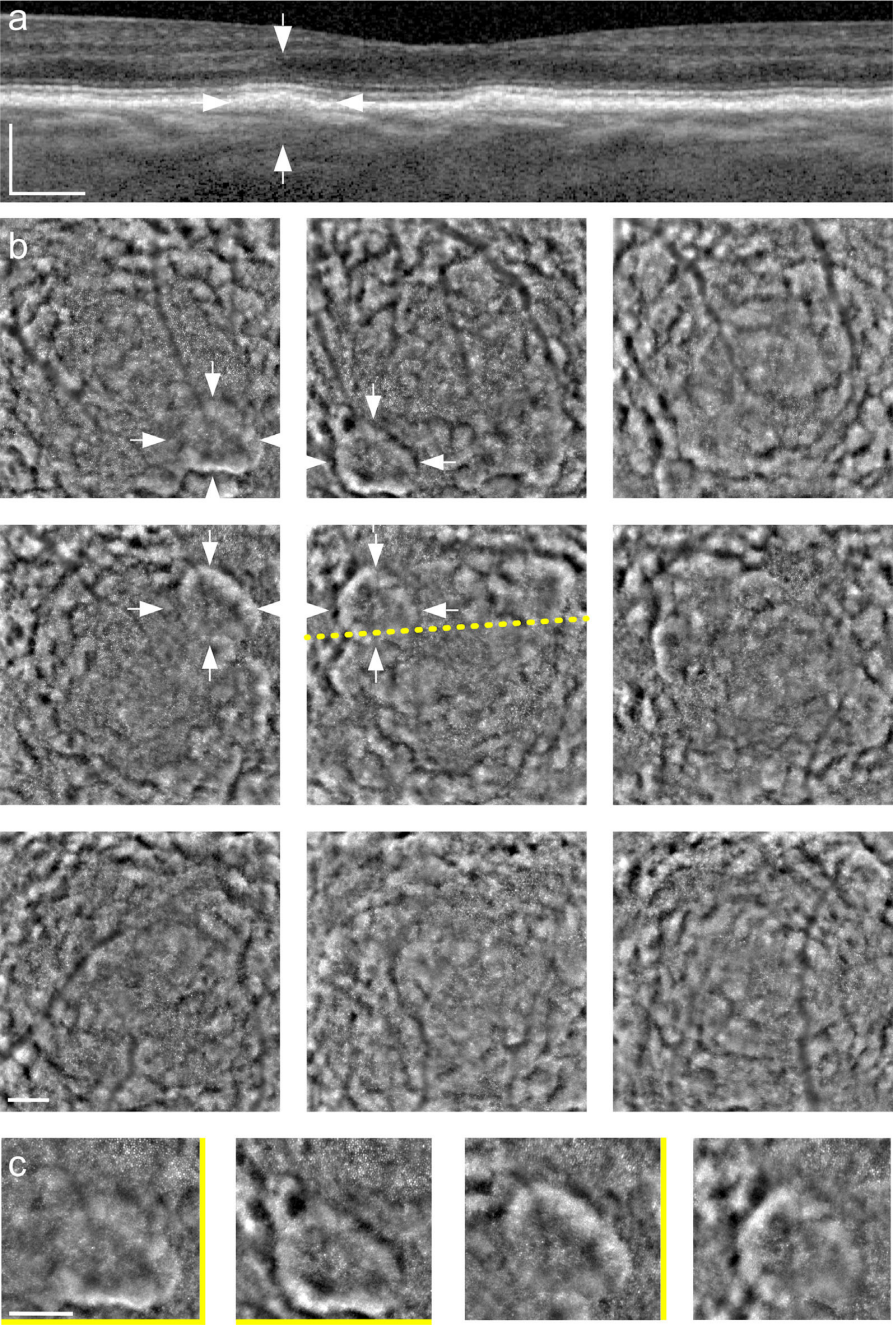
Source images. OCT b-scan (a) through the region indicated with the yellow dotted line on the FIAO images (b). FIAO images (4° × 4° field of view) were acquired from nine gaze positions, including the preferred fixation locus (b, *center*, taken to be 0,0) and locations separated by 2° in superotemporal (b, *upper left*), superior (b, *upper center*), superonasal (b, *upper right*), temporal (b, *center left*), nasal (b, *center right*), inferotemporal (b, *lower left*), inferior (b, lower center), and inferonasal (b, *lower right*) to central fixation. White arrows denote an individual drusen (a, b); note how the contrast of this druse varies among the four gaze positions that capture its extent (zoomed region in (c), where yellow bars indicate gaze orientation; note that the final image in that row has no yellow bars because gaze was directed straight ahead [i.e., fixation at 0,0]). Participant was a 71-year-old woman whose fundus photo was graded as intermediate AMD. See Supplementary Animation S1 for longitudinal animation. *Scale* *bars*: 200 µm.

Participants were imaged with identical commercial FIAO systems (device version: rtx1-e; software version: AOImage 3.4; Imagine Eyes, Orsay, France) deployed at each site. FIAO was performed under mydriasis and cycloplegia with one drop each of tropicamide 1% ophthalmic solution and phenylephrine 2.5% ophthalmic solution. After dilation, several images of the central macula were obtained with the FIAO camera focused on the cone photoreceptors. The FIAO imaging location was guided by patient fixation on a target consisting of a yellow cross that was internal to the FIAO system. Participants were instructed to point their eyes at the cross, and it was repositioned to acquire 4° × 4° field of view images at nine locations surrounding the fovea (see Fig. 1 and Supplementary Animation S1). Each position was separated by 2°, providing 25% to 50% overlap between adjacent images.

### Image Processing and Analysis

We evaluated statistical combinations of FIAO images from different gaze positions. The principle is to highlight the pixels showing variability within each set of registered overlapping images. To carry out these computations, the images first needed to be registered. Registration and all additional image processing steps were carried out using a custom MATLAB script (R2020a; The MathWorks, Inc., Natick, MA, USA). This process began by first spatially filtering the images using a low-pass spatial filter with a radius of 25 pixels to remove noise and some higher frequencies. Image registration was then carried out with the filtered images using FFT-based phase correlation and a Matlab implementation of Fourier-Mellin image registration.^23^ The Fourier-Mellin transform provided rotation, scale, and translation; here, only translation and rotation were used. Registration was carried out between all pairs of images and the final position was taken to be the average offset given from each of the image pairs.

**Figure 2.**
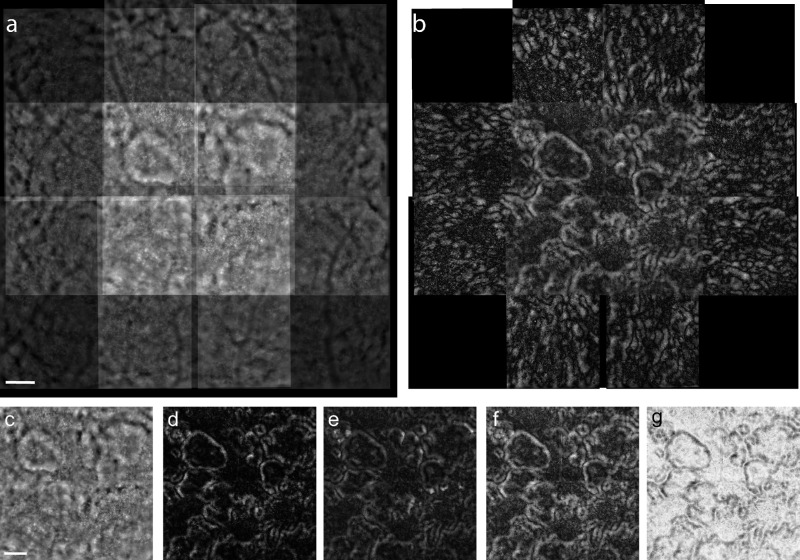
Fusion of registered images to enhance drusen contrast. The nine FIAO source images of Figure 1b were registered and combined. The resulting sum image is shown in (a) to demonstrate that the most overlap occurs within the approximately central 4° × 4° area corresponding to the image with gaze straight ahead. Computing the standard deviation for each pixel in (a) results in the image shown in (b). Note that the corners are blank in (b) because of only one image contributing to those areas; additional images (c–g) show only the central 4° × 4° region. The contrast of the drusen is diminished if the images are averaged (c) but computing the variance (d) and covariance (e) each enhances the margins around the drusen. Drusen contrast appears greatest when each pixel of the composite image uses the maximum SD value found between any of the two image pairs combined (f) and is sometimes most visible in an inverted contrast image (g). *Scale* *bars*: 200 µm.

Once all offsets were computed, the central image (i.e., with coordinates 0,0) was taken to be the center of a new image stack and its bounds were expanded to cover the entire area of the registered image stack. Using the registration data, each of the native (i.e. unfiltered) images was then translated and rotated and placed into the new image stack. Rather than set the “background” of these images to be 0 or 1, pixels not containing data were set to “not a number” (i.e., NaN), so that computations could be performed in Matlab that did not take those pixels into account. This was accomplished with the built-in Matlab functions such as nanstd and nanmean. We explored different methods for enhancing the contrast of the drusen, for example, by computing the standard deviation (SD) or difference between each overlapping image, some of these are shown in Figure 2. We found that drusen contrast appeared subjectively highest when we created an image using the value of the largest SD found between each pair of images (see Fig. 2f). In some cases, inverting the contrast of the images provided better subjective contrast (see Fig. 2g).

### Multimodal Registration

We used the vessel landmarks in the FIAO montage to determine the precise position of the FIAO image area on the color fundus and SLO images. Multimodal image registration was carried out using i2k retina (DualAlign LLC, Clifton Park, NY, USA), using the color fundus image as the target image for the infrared SLO image. All registered en face modalities were then imported into a multilayer image and scaled up to the size of the FIAO images in Adobe Photoshop (Adobe Systems Inc., San Jose, CA, USA). Vessel landmarks were then used to manually align the FIAO images to the clinical images.

### Drusen Quantification and Comparison Between Gaze-Dependent Images and Color Fundus

To evaluate the efficacy of our gaze-dependent FIAO images to detect drusen, we compared the drusen that could be detected and measured in gaze-dependent images to the historical gold-standard of color fundus images. We were interested in both the number of drusen that could be detected in each imaging modality and their sizes. We chose to quantify drusen across four image pairs, one of which contained intermediate to large drusen, one small to intermediate, and two containing smaller drusen. Five image graders participated to compute statistics on intergrader variability.

**Figure 3.**
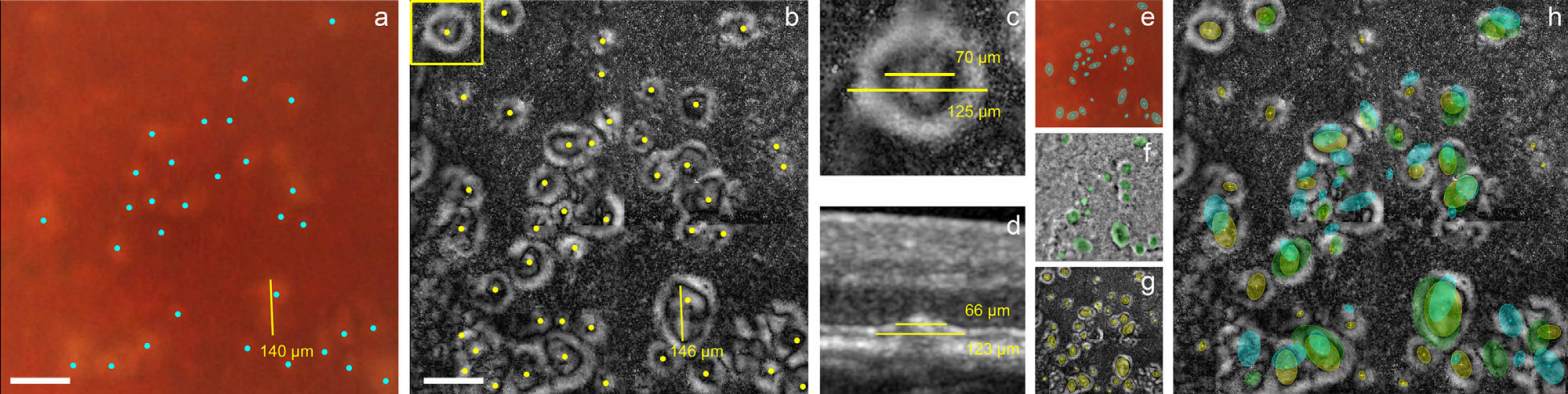
Drusen quantification. Drusen were counted on the color fundus (a; cyan dots) and gaze-dependent (b; yellow dots) image pairs and their diameters measured (yellow lines in a and b). Boxed area in (b) indicates location of zoom shown in (c), with inner and outer drusen diameters measured, and the corresponding OCT b-scan through this region (d), with drusen diameters measured at halfway to summit and at base, matching the inner and outer diameters measured in gaze-dependent images. Drusen area was quantified by graders drawing ellipses on the color fundus (e; cyan ellipses), native AO (f; green ellipses), and gaze-dependent images (g; yellow ellipses). All ellipses are overlaid together on the gaze-dependent image in (h), for one set of images from one grader, to visually appreciate variability in the extent of drusen detected in each modality. *Scale bars*: 150 µm.

Overlaid color fundus and gaze-dependent images were opened in Photoshop. First, on the color fundus photograph, we marked each spot we considered to be a druse with a white dot (see Figs. 3a–d). Then, we switched to the gaze-dependent image and marked each drusen with a yellow dot. We then increased transparency until we could see the overlay of the two images with their white and yellow dots. We checked that each white dot was also tagged with a yellow dot (to verify that all drusen visible with color fundus are also visible with gaze-dependent). We next counted the number of white dots versus yellow dots. We therefore measured the following parameters: (i) the number of additional drusen detected in gaze-dependent images compared to color fundus and (ii) any discrepancies of drusen that were visible in color fundus but not visible in gaze-dependent.

The next step was to measure the drusen size in the images. This was done by opening the color fundus and gaze-dependent image pairs in ImageJ and using the ruler tool to mark the diameter of a selection of drusen (scale factor: 1 pixel = 0.77 µm). We marked five drusen in each image in this way, making sure to pick the largest, the smallest, and three others that were most representative of the general drusen size in that image. This gave us a measure of the mean drusen size in each image and the range. On drusen that were visible in both modalities, measuring inside the gaze-dependent annulus gave a size closer to the color fundus measured diameter than if we included the white annulus in the diameter. This suggests that the inner dark area corresponds to the drusen summit whereas the white annulus corresponds to the drusen slopes. To confirm this, we checked against diameters measured on OCT. If we measure the drusen on OCT as the black drusen interior, it matches the inner diameter of the gaze-dependent annulus and the color fundus measurements. If we measure the width of the black drusen interior plus its white slopes on OCT, at mid-height, this matches the outer diameter of the white annulus plus its black interior in gaze-dependent images (see Figs. 3c, 3d). The ratio of inner to outer diameter of the annulus in gaze-dependent images and the drusen center to center plus slopes, that is, the dimensionless measure, gives approximately the same ratio in OCT and gaze-dependent images. To complete the quantification, five different graders independently and blindly repeated the process described above (i.e., marking visible drusen with dots on the three color fundus versus gaze-dependent pairs, measuring diameters on five drusen from each image including largest, smallest, and three in between) to assess intergrader variability.

Finally, we also quantified drusen using a custom made Matlab script that allowed for ellipses to be drawn overtop of the images (see Figs. 3e–h). For this procedure, each of the five graders placed an ellipse over every drusen that they could detect in each of the four sets of images used for the previous procedures. Here we also added in the native FIAO images, so that we could compare the difference between the number and size of drusen that could be detected also on the native FIAO image. For this procedure, the graders drew ellipses on each image modality in turn, starting first with the color fundus (e.g., Fig. 3e), followed by the native FIAO image (e.g., Fig. 3f) and then the gaze-dependent image (e.g., Fig. 3g). We compared the number of drusen detected on each image and took the major axis diameter of each ellipse as the diameter of the drusen.

### FIAO Directional Imaging

To better understand the light-tissue interaction causing gaze-dependent variability, we compared it to FIAO directional imaging in a few participants. In gaze-dependent imaging several images are obtained at different gaze positions, with each having a different field of view (see Fig. 1). Since the operator takes care to ensure that the illumination beam is centered on the pupil for each image acquisition, gaze-dependent FIAO imaging does not change the position of the illumination beam and it remains centered on the pupil of the eye. In contrast, directional FIAO^24^ imaging capitalizes on the variations in contrast that are introduced in FIAO when displacing the illumination beam with respect to the pupil of the eye. Images are obtained while the fixation target and thus field of view remain fixed and the operator purposely shifts the position of the illumination beam so that it does not remain centered on the pupil of the eye, which modifies the angle of incident light.

The full details of the directional imaging method are provided elsewhere^24^ and will only briefly be described here. First, a fovea-centered image was acquired with the illumination beam centered in the pupil. Then, four additional images were acquired after the position of the illumination beam with respect to the pupil of the eye was displaced in the cardinal directions (up, right, down, and left). The position of the illumination beam was controlled by the operator by using the joystick to displace the green cross that denotes the illumination axis, displayed on the live anterior segment image, to the desired positions within the pupil of the eye. This resulted in an approximately 2° variation of the angle of incidence on each side, that is, 4° in total. All five images were of the same fovea-centered field of view because the fixation target remained fixed for all acquisitions. The five images were then aligned using i2K retina. The presence of parallax, manifesting as a shift of the vascular shadows overlying the RPE/photoreceptors, that is a function of the angle of incident light was visually checked. The directional image was then generated using the standard deviation function of the Z projection of the registered image stack in ImageJ.^25^

## Results

Color fundus photos of sufficient quality for grading on the Beckman clinical classification scale^4^ were acquired from 169 of 182 AMD eyes. These were graded on the Beckman scale as follows: normal aging changes (n = 7), early AMD (n = 12), intermediate AMD (n = 108), or late AMD (n = 42). SDDs were detected on SD-OCT images of 27 eyes. FIAO images could be obtained in all eyes and the gaze-dependent registration procedure was successful in 90% of these (n = 163); when registration failed it was most often due to inaccurate fixation (n = 6) or poor image quality (n = 6).

With gaze-dependent imaging, drusen had a slightly irregular annular form, with a center that was less reflective than the periphery. In all drusen observed, native FIAO images (i.e., standard images generated using the built-in processing of the rtx1 device, without further postprocessing) showed systematic variations in brightness and contrast. In general, when a druse was closer to the edge of the image field of view (FOV) than the center, the margin of the druse that was closer to the edge of the FOV was brighter and of higher contrast than the opposite side that was usually low contrast. In Figure 1, the white arrows mark a single druse to illustrate how the contrast of the margin of that druse varies as gaze position was changed. Figure 1c shows a zoomed view of this druse, along with yellow bars denoting the gaze direction, to show how the various margins of this druse varied with gaze position. The images in Figure 2 show the native FIAO images from Figure 1c registered. It can be seen here that the druse mentioned above can now be rendered to be nearly isometric in contrast across the entire margin of the druse when the images are fused using statistical operations that pinpoint pixels that vary in intensity with gaze position. The standard deviation (Fig. 2b), variance (Fig. 2d), and covariance (Fig. 2e) all enhance the contrast isometrically around the drusen, whereas summing the images (Fig. 2a) or averaging them (Fig. 2c) tend to diminish the contrast. We found the highest contrast was obtained when we assigned each pixel the value of the maximum SD found for that pixel between any two image pairs (Fig. 2f). Gaze-dependent imaging was reproducible, as shown by the comparison of pairs of images taken at intervals of a few days. There was, however, some intersession variability because of the residual background signal due to the cone mosaic.

**Figure 4.**
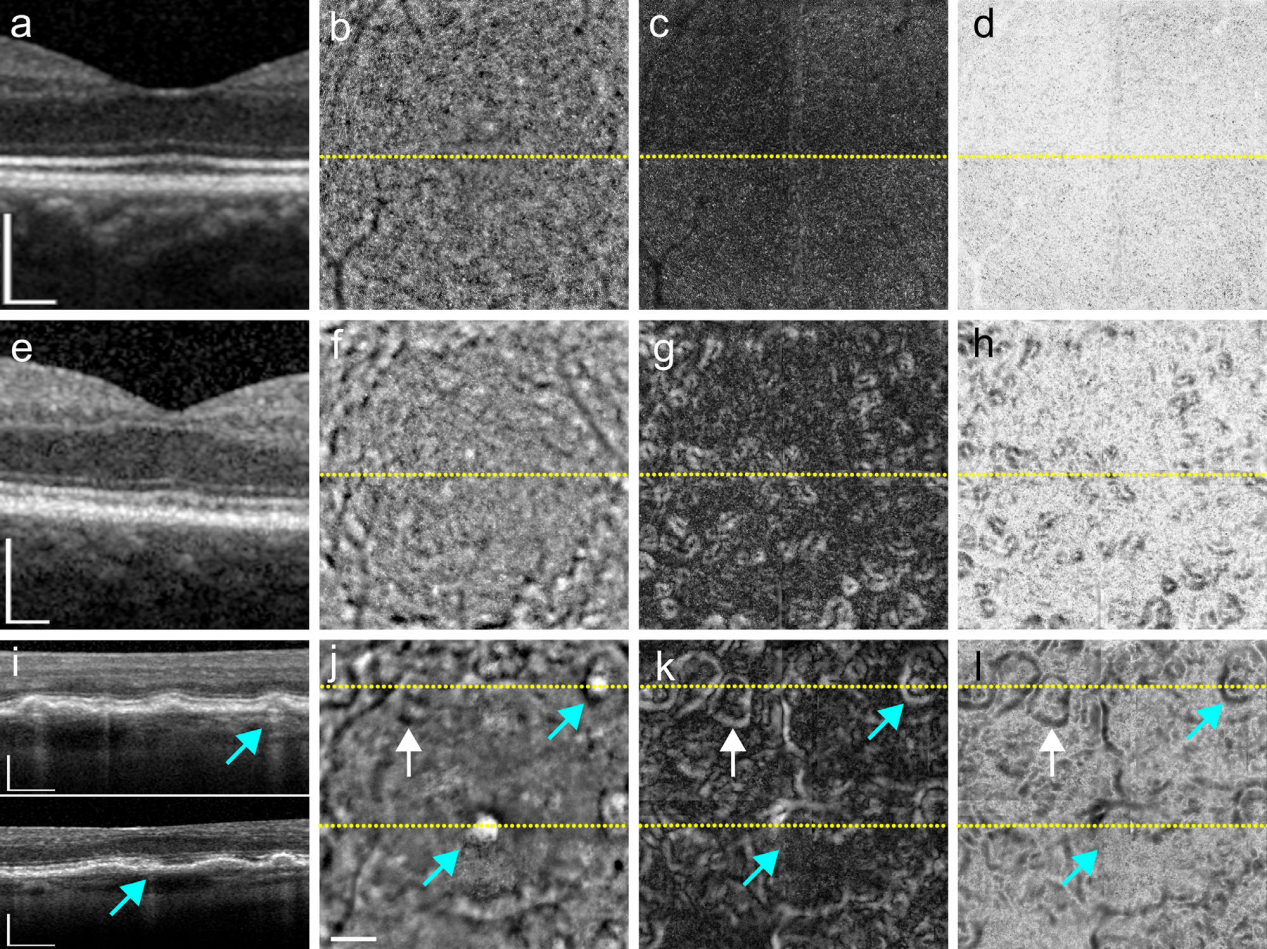
Gaze-dependent images compared to OCT in normal eyes, confluent drusen and geographic atrophy. OCT (*first column*) and raw FIAO images from the (0,0) location (*second column*) shown in comparison to the gaze-dependent images in standard contrast (*third column*) and inverted contrast (*fourth column*). Dotted lines in b–d, f–h, and j–l denote the position of the OCT b-scans shown in the first column, respectively. The younger cases (*top row*; 26 years old) showed no gaze varying structures (c, d); only a small cross shaped artifact centered on the image can be noticed that is likely due to minute residual errors in registration. The second row of images from a 74-year-old patient with AMD (e–h) shows that even in the cases of many small confluent drusen, each individual druse can be delineated in the gaze-dependent images (g, h). The *bottom row* shows coalescent drusen in a 77-year-old with AMD (white arrow), and two small spots of atrophy (denoted by the blue arrows). Although these small atrophic areas are visible as hyper-transmission in the corresponding OCT b-scans (i) and saturated bright pixels in the FIAO image (j), their appearance in the gaze-dependent images differs, with the upper one resembling a druse (k,l; upper blue arrows), whereas the lower one is not visible (k; lower blue arrows). *Scale* *bars*: 200 µm.

Figure 4 shows a representative spectrum of gaze-dependent images from various ages. On gaze-dependent images most control eyes younger than 30 years old showed a diffuse background signal reflective of the cone mosaic with no gaze-dependent structures (Figs. 4a–d), while drusen became apparent in images from older eyes (Figs. 4e–l). With increasing size, the appearance of drusen changed. Small drusen (<63 µm) had bright margins with a dark center, while intermediate to large drusen (>63 µm) occasionally contained additional bright regions within the center (e.g., see druse shown in Fig. 3c). Within closely apposed drusen (i.e., confluent drusen whose margins were adjacent), the margins of each individual druse could usually still be clearly discerned (e.g., Figs. 4k, 4l).

### Drusen Quantification and Comparison to Color Fundus Images

**Figure 5.**
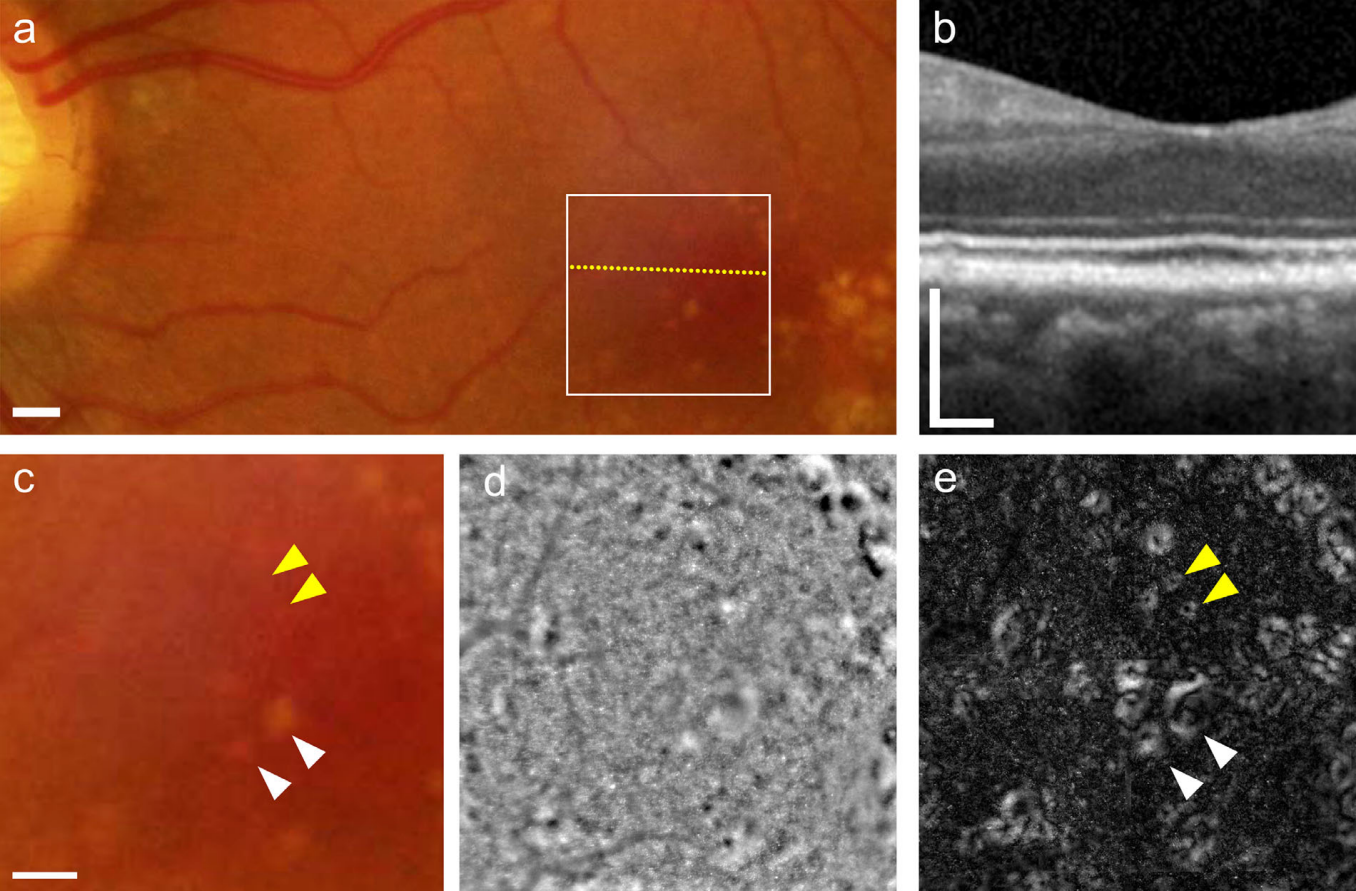
Multi-modal imaging of the macula of a 52-year-old woman with drusen. Color fundus photo (a) and OCT (b); yellow dotted line in (a) denotes location of OCT b-scan shown in (b) whereas the white square denotes the location of the region of interest shown in (c–e), where (c) is a zoom on the color fundus, (d) is the native FIAO image when gaze was directed straight ahead, and (e) is the gaze-dependent image with enhanced drusen contrast. Some larger drusen were seen temporal to the region of interest in (a). The gaze-dependent images revealed some drusen seen in the clinical images (corresponding white arrowheads in c and e), but also revealed the presence of putative small drusen that are not seen in the clinical images (compare yellow arrowheads in c and e). OCT (b) showed minimally detectable drusen across this area. *Scale bars*: 200 µm.

Careful comparison between each gaze-dependent image and its corresponding color fundus image revealed many drusen that appear in both (compare drusen denoted with white arrowheads in Fig. 5c to Fig. 5e). The gaze-dependent image also shows several smaller structures (<25 µm in diameter) that were not visible in the clinical images (compare drusen denoted with yellow arrowheads in Fig. 5e and Fig. 5c). It is of interest to note that on single images, these small lesions had a brighter side when closer to the edge of the FOV of the image, similar to typical drusen, and these small lesions coexisted with drusen. Given the phenotypic continuum between these small features and the larger drusen seen in older subjects, we hypothesize that most of the small bright ring-like contours are drusen. However, additional studies are necessary to distinguish physiological (normal aging-related) drusen from age related maculopathy drusen.^26^ Prospective studies using gaze-dependent FIAO imaging may help to answer this question. These minute drusen manifest in most cases as a central dark spot surrounded by a bright annulus; this is because the intensity of the pixels in the central portion remained relatively constant across gaze positions and, hence, generated no signal when we computed the maximum SD across images. We also occasionally detected tiny putative drusen with no central dark region that were uniformly bright.

Drusen quantification based on linear measurement of drusen diameter revealed (i) a clear increase in number of drusen detected with gaze-dependent images compared to color fundus (average: 130% for medium to large and small to medium drusen; 250% for small drusen; intergrader variability 30%); and (ii) all drusen visible in color fundus were detected in gaze-dependent images, even those of larger size, with the exception of two structures that we had marked as drusen based on the color fundus image but that were in fact identified as small atrophic areas on comparison with OCT. Atrophic areas are usually not visible in gaze-dependent imaging because they are not gaze-varying structures (see nuanced details for areas of small atrophy within drusen in section on atrophy below and Fig. 4). It should be noted the importance of the order of grading for this type of manual analysis because the drusen were marked on color fundus first and then on the gaze-dependent images. If, after grading, the color fundus and gaze-dependent images are placed side by side, with careful searching, nearly all of the drusen identified in the gaze-dependent images are weakly visible in the color fundus but are not picked up if viewing color fundus alone because of their extremely low contrast. The size of drusen as measured by linear measurements in gaze-dependent imaging ranged from 11 µm to 320 µm (n = 75). Intergrader variability on diameter measurement was greater on large drusen (30% variability) compared to small drusen (6% variability).

**Figure 6.**
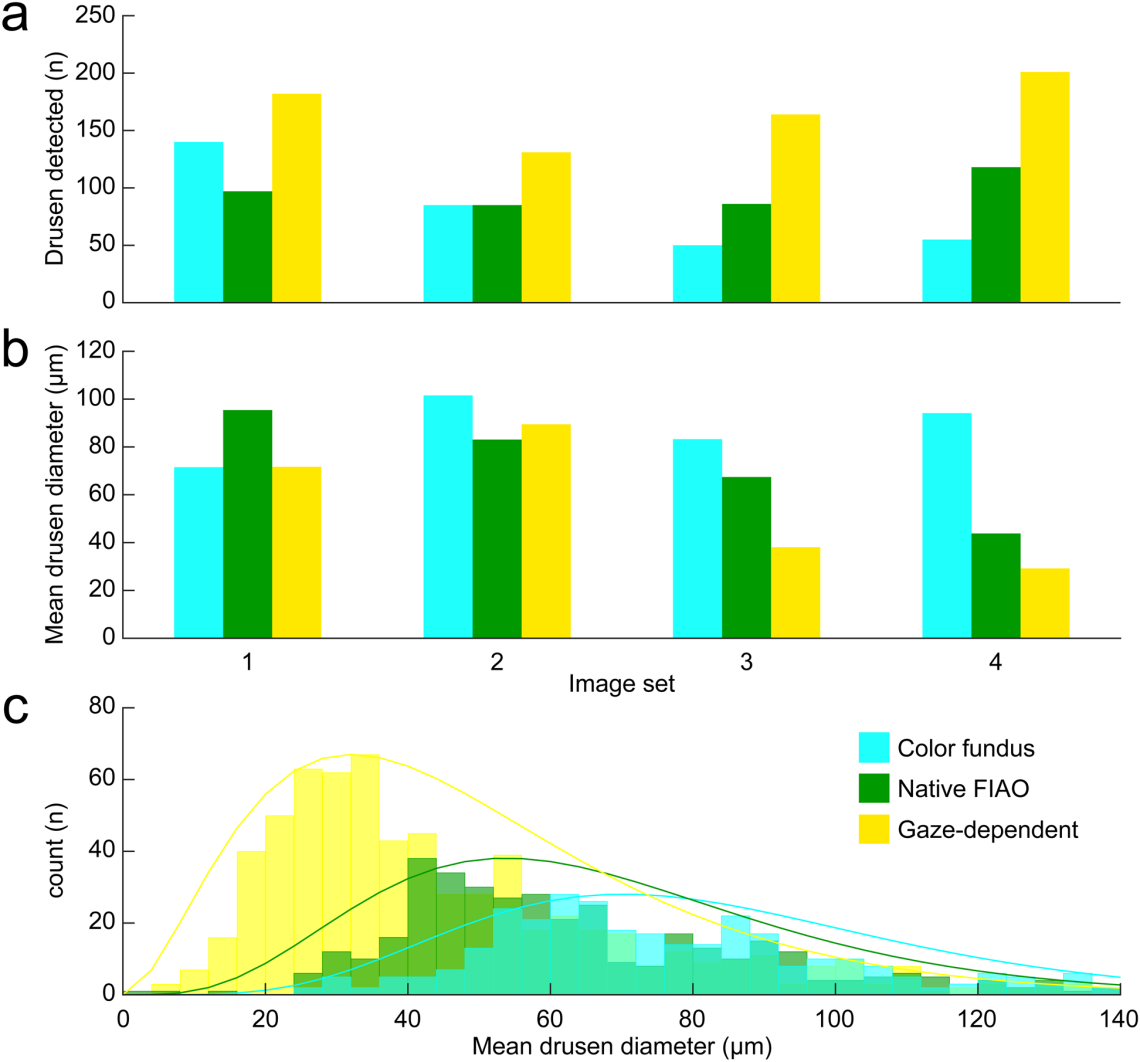
Smaller drusen were detected more frequently on the gaze-dependent images compared to color fundus and native FIAO images. Using the ellipse-based manual annotation method (see Figs. 3e–3h), we evaluated the number of drusen that could be detected across four sets of color fundus (cyan), native FIAO (green), and gaze-dependent images (yellow). In all cases, the gaze-dependent images revealed more drusen (a). Comparing the mean drusen diameter computed across the image pairs (b), we see that when the mean drusen diameter was measured to be greater than 40 µm on the gaze-dependent images, the average drusen diameter was similar to that measured with the other modalities (i.e., image sets 1 and 2). However, when mean drusen diameter in the gaze-dependent images was found to be less than 40 µm (i.e., image sets 3 and 4), the difference between the mean diameter measured on the color fundus was substantially greater, nearly twice the average measured for the gaze-dependent images with the native FIAO falling in between. Histograms in (c) show that gaze-dependent images revealed many more drusen less than 40 µm in diameter than could be detected in the native FIAO images or on the color fundus image.

Drusen quantification using the ellipse drawing method produced similar results to that seen for the linear measurements (Fig. 6). This analysis also showed that the number of drusen detected when comparing the color fundus, native FIAO and gaze-dependent images was always greatest for the gaze-dependent images. Mean drusen diameter measurements were comparable between the color fundus, native FIAO, and gaze-dependent images when the mean drusen diameter was measured to be greater than 40 µm in the gaze-dependent images (i.e., image sets 1 and 2 in Fig. 6b). However, when mean drusen diameter in the gaze-dependent images was less than 40 µm (i.e., image sets 3 and 4 in Fig. 6b), the difference between the mean diameter measured on the color fundus was substantially greater, nearly twice the average measured for the gaze-dependent images, with the native FIAO falling in between. This difference was due to many more drusen being detected with diameters of less than 40 µm in the gaze-dependent images compared to the other modalities, as shown in the histograms in Figure 6c.

### Gaze-Dependent Images of SDDs

**Figure 7.**
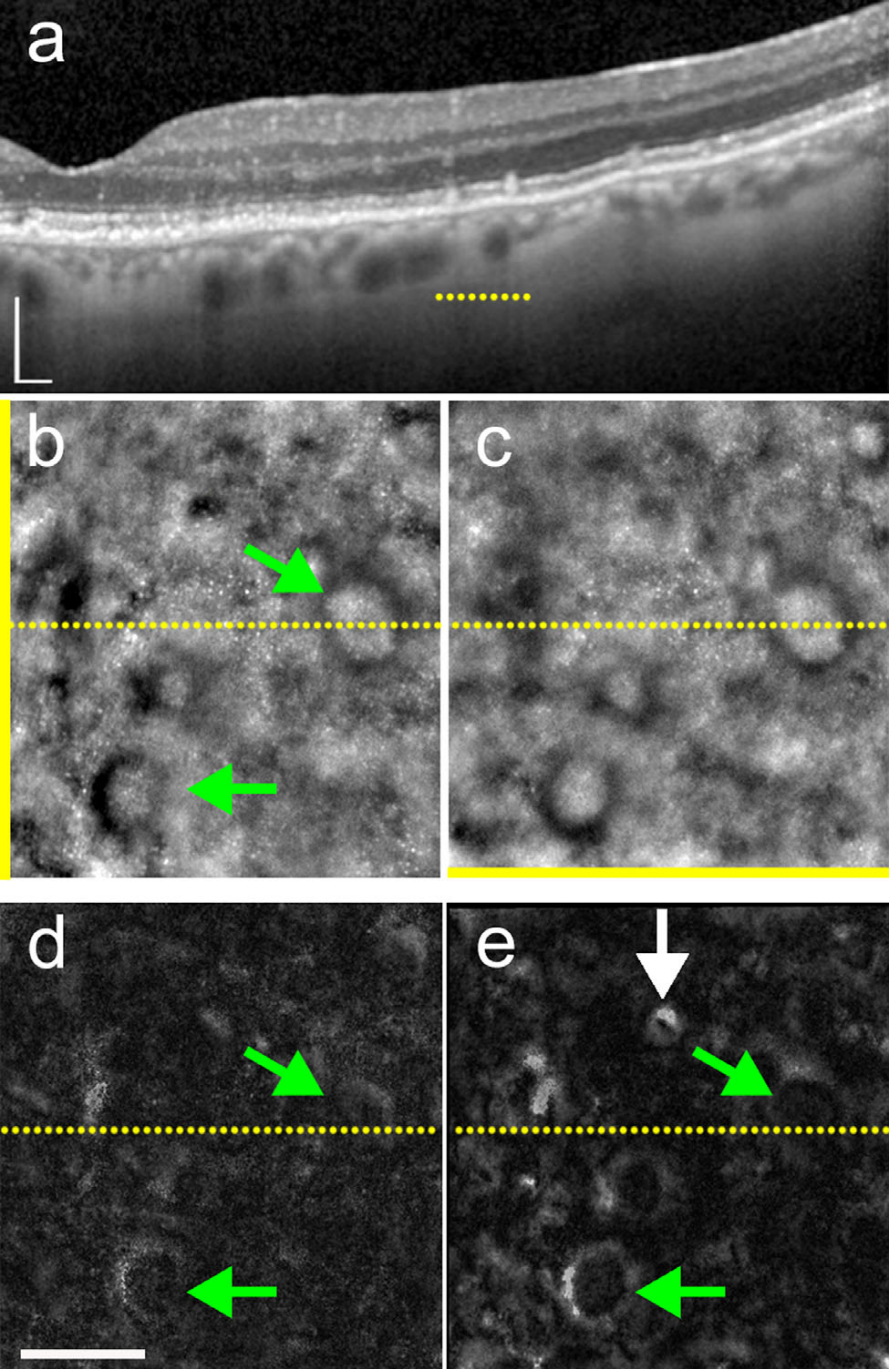
Gaze-dependent images of subretinal drusenoid deposits. OCT (a) and FIAO images (b, c) with yellow bar indicating gaze orientation. Extent of OCT line and location on (b) and (c) is denoted by the dotted yellow lines. Directional (d) and gaze-dependent images (e) showing variable appearance of stage 3 SDDs (two SDDs are denoted with green arrows). Some SDDs exhibited variability with gaze position and thus had higher contrast in the gaze-dependent images (e.g., SDD denoted by lower green arrow), while others varied less and were not high contrast in the gaze-dependent images (e.g., SDD denoted by upper green arrow). Note also the patchy bright signal surrounding SDDs in the gaze-dependent images. A druse within this field of view appears high contrast in the gaze-dependent image (white arrow, e). *Scale bar*: 100 µm.

In native FIAO images, stage 3 SDDs typically appeared as dark rings (Fig. 7); comparison to the OCT suggests that the deposit is in the center of the ring and that the ring may indicate deflected photoreceptors. Compared to drusen, gaze-dependent images of SDDs showed similar variations in contrast and brightness when close to the edge of the FOV but of an opposite sign (i.e., the contrast polarity is inverted). In SDDs the margin of the SDD closer to the edge of the FOV became darker, whereas the opposite margin became brighter. Because our procedure enhances this variability irrespective of the sign of these changes, on gaze-dependent images SDDs appeared also as a bright ring (because those areas have a high SD among the different gaze positions). In this regard, it is of interest to note that a minute druse visible adjacent to the SDDs is of higher contrast than the SDDs (white arrow in figure panel 7e). SDDs also showed poor contrast because of gaze-dependent variability in the contrast of the background, leading to a diffuse linear brightness. Thus SDDs are easier to recognize in native FIAO images than on gaze-dependent images.

### Gaze-Dependent Images With Geographic Atrophy

We also examined 42 eyes with RPE atrophy. RPE atrophy most often manifests as regions of hyper-reflectivity in FIAO that exhibit little variation in contrast with gaze position, so were usually not enhanced in gaze-dependent images. However, in a few cases we noticed that minute areas of atrophy (e.g., blue arrows in Fig. 4) could have variable appearance in gaze-dependent images (Figs. 4k, 4l), with either a drusen-like appearance (upper blue arrow) or were not visible (lower blue arrow). For larger atrophic areas, the strong signal from the GA region usually dominates the native FIAO image, reducing image contrast across the entire field of view. When these types of images were registered with adjacent fields of view that did not contain atrophy to generate gaze-dependent images, the large differences in image brightness and contrast between the areas containing atrophy and those free from atrophy resulted in images with clear well-defined boundaries between the atrophic and nonatrophic images but no contrast enhancement of the GA.

### Comparison Between Gaze-Dependent Imaging and Directional Imaging

**Figure 8.**
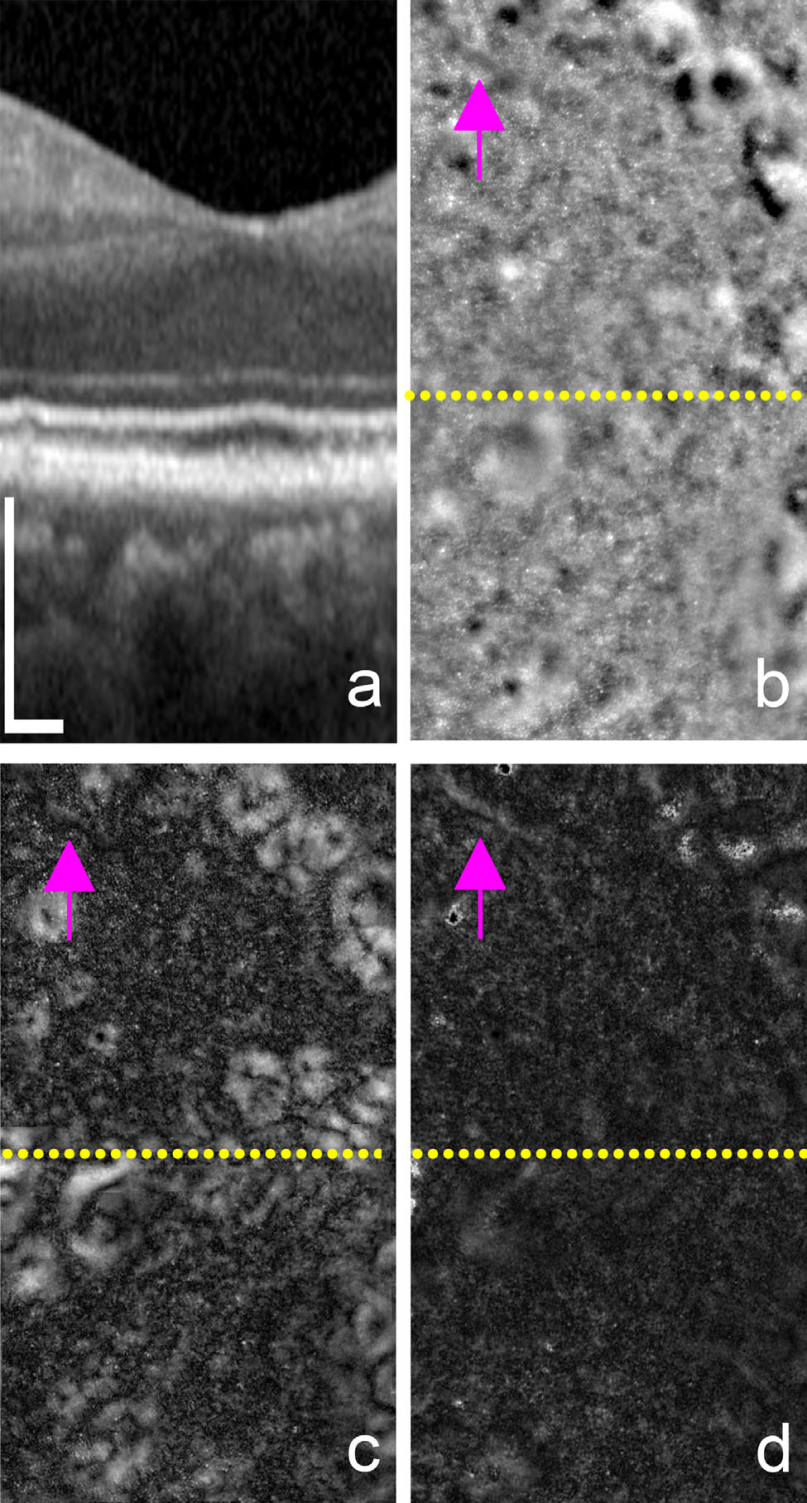
Comparison between gaze-dependent and directional images. OCT scan across the fovea (a) and native FIAO images (b) are shown in comparison to the gaze-dependent (c) and directional (d) images that were taken using the protocol described previously by Miloudi et al.^24^ Location of the OCT scan on the images in (b-d) is denoted with the dotted yellow line. Gaze-dependent images reveal the drusen structures with high-contrast (c) while directional images lack high contrast features across most of the image (d). The exception is the upper right corner of (d), where there is some increased signal in the directional images; note that these features are also very high contrast in the native FIAO image (b). Another difference is the appearance of the vessel structure (denoted by the pink arrows in b, c, and d). Note that parallax due to the different illumination angles causes the vessel position to shift with illumination angle in the directional images, resulting in a higher SD and higher contrast vessel (d, arrow). The patient was 52 years old at the time of imaging. *Scale bars*: 200 µm; horizontal scalebar in a applies to (b–d).

We compared gaze-dependent imaging to directional imaging in three cases. In all cases, we observed that drusen showed a stronger variability with gaze than that related to changing the direction of light incidence, resulting in gaze-dependent images consistently producing higher contrast images of the drusen (Fig. 8). Gaze-dependent images also produced higher contrast for SDDs than the directional images (see Fig. 7d).

### Longitudinal Gaze-Dependent Imaging

**Figure 9.**
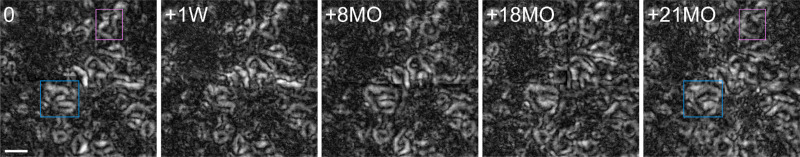
Evolution of drusen in gaze-dependent imaging demonstrates repeatability and reveals subtle changes over time. Gaze-dependent imaging at several time-points of drusen in the fovea in the right eye of an 87-year-old woman. Nearly the same pattern was seen across these images that were taken over the course of 21 months. Text in upper left corner denote intervals between imaging that occurred at time point zero (far left) and in follow-up at one week, eight months, 18 months, and 21 months. Some small changes exist, but these are best revealed when the sequence is animated and are difficult to appreciate by eye. Two sections that changed between the first and final timepoint are highlighted with the pink and blue boxes. Within the area denoted by the pink box, a few drusen at the first timepoint appear to merge into a single druse by the final timepoint. In the area denoted by the blue box, similar merging of apposed drusen can be seen; here we see what appear to be several drusen in the process of merging. An additional tiny druse in the lower left corner of the box stays distinct from the others for the first four timepoints but it then appears to begin to merge with the others at the final timepoint. *Scale bar:* 200 µm.

To evaluate changes over time, in 83 subjects we generated gaze-dependent images at several intervals ranging from weeks to several months. Figure 9 demonstrates that we can reliably observe comparable patterns of drusen distribution over time, with careful inspection of local regions (e.g., pink and blue boxed areas) revealing subtle changes. In larger drusen, we observed expansion and apparent fusion of adjacent drusen (see Visualization 1). These changes were best appreciated when viewing the images as an animation rather than in the static images. We also observed some of the hard drusen that appeared in the first timepoint take on the more characteristic darker center/brighter surround appearance at the later timepoints as they expanded in size (see arrowheads in lower right quadrant of Visualization 1). This suggests that the smallest structures we detected are early drusen that are not readily detected using other imaging methods.

## Discussion

Here we show that high-contrast images of drusen can be achieved by statistical combination of overlapping FIAO images. Gaze-dependent FIAO images showed nearly isometric delineation of contiguous drusen margins, even in the case of confluent drusen and revealed some tiny putative drusen that were not visible in clinical images. Applying this technique to images from younger participants reveals no gaze-varying structures, whereas in aging and AMD we observed a spectrum of changes, including the detection of minute drusen in healthy aging. Our drusen quantification results clearly show that smaller drusen (<30 µm in diameter) are much more readily detected in gaze-dependent images than in fundus photos or in the native FIAO images themselves. This finding is consistent with previous work that compared histopathology to the clinical appearance of drusen. Sarks et al.^26^ showed that minute drusen are common in normal aging; however, only drusen deposits larger than 25 to 30 µm in diameter were detected clinically in fundus photographs.

We compared the present technique to our previously demonstrated directional imaging that varies the illumination beam in the pupil to show that images obtained from each method do not reveal drusen with the same contrast. We also showed that gaze-dependent images are highly repeatable across imaging sessions and can be used to track the fine scale progression of drusen evolution over time. Finally, we show time-lapse images demonstrating both reproducibility of the technique and subtle changes over time in the case of slowly progressing drusen (Fig. 9) and an example of substantial progression where drusen are seen to emerge, expand, and coalesce over time (Visualization 1). Gaze-dependent imaging produces high-contrast images of drusen, but contrast is not substantially enhanced for SDDs or pigment mottling, which leads to the possibility that this approach may be of interest to specifically highlight drusen within a complex mix of fundus changes.

To our knowledge this is the first observation of this process. However, a similar phenomenon of gaze-dependent variation of the dark ring around SDDs was reported using SLO IR imaging.^27^ The light-tissue interaction at the origin of the present phenomenon remains speculative, and both the optical properties of the deposits and their effects on the photoreceptors and surrounding tissue must be considered. To progress on this point, we evaluated whether gaze-dependent imaging could be similar to directional imaging. Misalignment of cone photoreceptors caused by mechanical disruption from drusen may possibly cause some backscattered light to be waveguided away from the pupil by cones on the way out of the eye. Thus cones opposed to drusen that are no longer pointing toward the center of the pupil may differentially waveguide some backscattered light leading to variations in drusen contrast with gaze position. The fact that the contrast modulation appears to happen at the margins of the drusen is consistent with a transition between normally pointing cones and cones whose directionality may be altered. However, directional imaging that displaces the illumination beam in the pupil but keeps the gaze fixed did not reveal the same contrast enhancement as that seen for gaze-dependent imaging. It therefore appears that the Stiles-Crawford effect may contribute to gaze-dependent imaging but does not fully explain the image formation mechanism. Another possible explanation is that the properties of the illumination beam may play a role, because variations in gaze position with a relatively narrow beam illumination may modulate the proportion of directionally back scattered versus multiply scattered light reaching the detector; the illumination beam diameter in the rtx1 device used here is 2.7 mm. Further study of this phenomenon using different illumination beam diameters is warranted to understand how illumination beam diameter may modulate the gaze-dependence of drusen contrast.

Although our sample of SDDs here was small, it is of interest to note that drusen and SDDs show differing patterns of gaze-related changes. This is likely due to the differences in the axial position of these structures within the laminar organization of the retina, with drusen beneath the photoreceptors potentially driving a photoreceptor-related contrast modulation mechanism that differs from the changes caused by SDDs. SDDs were well defined in the native FIAO images, so this difference may help differentiate drusen from SDDs in vivo and help guide future image processing for selective enhancement of specific features of interest. Interestingly, we found gaze-dependent structures at some distance from stage 3 SDDs, suggesting that this technique may also reveal diffuse changes that may be a harbinger of SDDs or drusen yet to come. Histology studies have shown that stage 3 SDDs may be associated with confluent stage 1 or 2 SDDs.^28^ It is therefore conceivable that the confluence of these subretinal deposits may also exhibit gaze-dependent variability that alters the visibility of stage 3 SDDs, but this requires further study.

The protocol we used here to sample the central macula with nine gaze positions is easy to implement in clinical imaging workflows and could be expanded with more images to cover a larger area. Although the FIAO commercial system used here has lower resolution than many research AOO instruments, this does not appear to be a drawback for gaze-dependent imaging of drusen. One limitation is that patients must be able to fixate somewhat accurately on the internal fixation target for these types of images to be acquired, although this is a limitation for all systems that require patient fixation to guide imaging location. Another limitation is that it becomes time consuming to perform such a procedure over larger areas of the retina; we limited our examples to a central 4° field, which takes approximately three to four minutes of acquisition time by a trained technician. On the other hand, the image processing is relatively fast (<3 minutes) and could be made even faster, so it could be implemented as a quick postprocessing step in commercial software and integrated into FIAO imaging workflows. Some aspects of our image processing could be further improved to mitigate the artifacts that occasionally arise in the images due to imperfect registration at the edges of some of the images. These manifest as a cross-shaped artifact in some of the gaze-dependent images and are more apparent in some images than others (e.g., these are seen readily in visualization 1 and in Figs. 4c and 4d).

Our quantification results suggest that gaze-dependent imaging may be useful to document the earliest stages of aging of the photoreceptor-RPE complex, particularly the smallest drusen (<25 µm), and to document the dynamics of early drusen progression. Although other methods for drusen contrast enhancement have been shown using clinical instruments, such as retromode scanning laser ophthalmoscopy, the resolution of these devices limits their sensitivity only to drusen greater than ∼25 µm.^29^ Several different methods for drusen quantification using clinical modalities have been proposed,^29^^–^^32^ and longitudinal data suggest that drusen volume quantification has predictive value.^9^^,^^10^ However, previous work has shown that automated OCT-based algorithms cannot detect the smallest drusen visible on color fundus photographs and that confluent drusen are challenging to segment.^29^ Our method may be complementary to these existing methods and may have a benefit for detecting and quantifying confluent drusen and the smallest sub-RPE deposits.

The methods for quantification we used here were sufficient to demonstrate the advantages of our approach for detecting the smallest drusen but are too tedious and time-consuming to be used regularly for drusen quantification. However, we are particularly interested in the potential for automated quantitative analysis of drusen from images such as those we show here, because we are confident that our images that clearly outline drusen boundaries will be more amenable to automated drusen segmentation than previous approaches that rely on subtle color or intensity variations that are difficult to reliably segment. Gaze-dependent FIAO images may therefore be useful for evaluating the efficacy of new treatments that aim to slow down or reverse non-neovascular AMD in its early stages. Ongoing work is underway to determine the efficacy and usefulness of gaze-dependent FIAO in other retinal and choroidal diseases, which may contribute to the understanding of the light-tissue interaction generating this phenomenon.

## Supplementary Material

Supplement 1

Supplement 2

## Supplementary Material

**Supplementary Animation 1**. Animation showing the principle behind the registration and composition of the gaze-dependent images (complementary to Figure 1). First, 9 FIAO images were acquired from 9 gaze positions in an 8-way connectivity. After the 8 neighboring images were registered, the maximum SD value between pairs of centrally overlaid layers was computed. Finally, pixels with the highest SD are used to generate a new composite image showing the drusen and their margins with high contrast.

**Visualization 1.** Substantial drusen progression over the course of three years. This animation demonstrates drusen progression at one-year intervals over a timespan of three years. Here we see marked progression, with some drusen expanding while others appear to coalesce. The dashed line denotes the extent of a druse at the first timepoint that is seen to expand on follow-up. The arrow points to a delineation between two drusen that disappears on follow-up, presumably because of fusion of these drusen into one larger druse. We also see regions where some tiny bright structures appearing in the first timepoint take on the more characteristic darker center brighter surround appearance at the later intervals; several of these are seen in the lower right quadrant. Two of these that appear to coalesce into a single druse at the final timepoint are denoted with arrowheads. The image field of view is 4° × 4°. The patient was 71 years old at the first imaging timepoint.
